# Predictive value of interim ^18^F-FDG-PET in patients with non-small cell lung cancer treated with definitive radiation therapy

**DOI:** 10.1371/journal.pone.0236350

**Published:** 2020-07-20

**Authors:** Nalee Kim, Jin Sung Kim, Chang Geol Lee

**Affiliations:** 1 Department of Radiation Oncology, Yonsei Cancer Center, Yonsei University College of Medicine, Seoul, Republic of Korea; 2 Department of Radiation Oncology, Samsung Medical Center, Seoul, Republic of Korea; Spedali Civili of Brescia, University of Brescia, ITALY

## Abstract

**Purpose:**

We evaluated that early metabolic response determined by ^18^F-fluorodeoxyglucose positron emission tomography/computed tomography (FDG-PET/CT) during radiotherapy (RT), predicts outcomes in non-small cell lung cancer.

**Material and methods:**

Twenty-eight patients evaluated using pretreatment ^18^F-FDG-PET/CT (PET_pre_) and interim ^18^F-FDG-PET/CT (PET_interim_) after 11 fractions of RT were retrospectively reviewed. Maximum standardized uptake value (SUV_max_) was calculated for primary lesion. Predictive value of gross tumor volume (ΔGTV) and SUV_max_ (ΔSUV_max_) changes was evaluated for locoregional control (LRC), distant failure (DF), and overall survival (OS). Metabolic responders were patients with ΔSUV_max_ >40%.

**Results:**

Metabolic responders showed better trends in 1-year LRC (90.9%) than non-responders (47.1%) (p = 0.086). Patients with large GTV_pre_ (≥120 cc) demonstrated poor LRC (hazard ratio 4.14, p = 0.022), while metabolic non-responders with small GTV_pre_ (<120 cc) and metabolic responders with large GTV_pre_ both had 1-year LRC rates of 75.0%. Reduction of 25% in GTV was not associated with LRC; however, metabolic responders without a GTV response showed better 1-year LRC (83.3%) than metabolic non-responders with a reduction in GTV (42.9%). Metabolic responders showed lower 1-year DF (16.7%) than non-responders (50.0%) (p = 0.025). An ΔSUV_max_ threshold of 40% yielded accuracy of 64% for predicting LRC, 75% for DF, and 54% for OS. However, ΔGTV > 25% demonstrated inferior diagnostic values than metabolic response.

**Conclusions:**

Changes in tumor metabolism diagnosed using PET_interim_ during RT better predicted treatment responses, recurrences, and prognosis than other factors historically used.

## Background

Fluoro-2-deoxy-D-glucose-positron emission tomography (FDG-PET) imaging has become an important and popular tool for determining the disease stage in patients with non-small-cell lung cancer (NSCLC). The National Comprehensive Cancer Network recommends the use of ^18^F-FDG-PET/computed tomography (CT) for the appropriate staging of lung cancer [1].

There are several roles of FDG-PET/CT in NSCLC, such as diagnosis, prognosis, and radiotherapy (RT) planning. Recent investigations have shown that FDG-PET/CT has more than 90% accuracy in diagnosis of malignant nodules, with a low false-positive rate [2]. FDG-PET also plays a significant role in nodal staging (accuracy 90%, sensitivity 79–85%, and specificity 87–92%) [3, 4] and distant metastasis detection, with previously unsuspected diagnosis of extrathoracic lesions in up to 10% of patients, beyond CT alone [5]. FDG-PET offers a benefit over conventional CT after treatment where, for example, although tumor shrinkage may be observed, inflammation and fibrosis after neoadjuvant chemotherapy or RT make assessment difficult [1].

In addition, FDG-PET plays an important role in target volume delineation of the gross tumor volume (GTV), for both the primary tumor and lymph nodes [6]. Its superior contrast between tumor and non-tumor tissue means that FDG-PET can also decrease inter-physician contouring variability, compared to delineation with CT alone [7]. It also greatly assists physicians in distinguishing the tumor tissue from atelectasis [8]. Therefore, a consensus report has been endorsed for target volume delineation using PET imaging [9].

Currently, chemoradiotherapy (sequential or concurrent) is considered as a standard treatment for locally advanced NSCLC. Despite the emergence of immunotherapy, targeted therapy, and new RT techniques, the prognosis of those patients remains poor. Therefore, the ability to identify non-responders during treatment, in order to change ineffective treatment early on, is very desirable [10]. Several studies have demonstrated interim PET (PET_interim_) metrics as a prognostic factor, but most of these included conventional three-dimensional conformal RT and various chemotherapy regimens, with varied timing of PET_interim_. Therefore, in this study, we focused on metabolic and volumetric parameters, which are easily accessible during RT, in patients treated with modern RT and certain chemotherapy regimens.

## Materials and methods

### Study population

Patients diagnosed with NSCLC who had undergone RT with PET_interim_ between March 2015 and January 2018 were enrolled. Patients were excluded if they underwent RT with preoperative aim (n = 7), if pre-RT FDG-PET/CT (PET_pre_) was not available or was performed at another institution (n = 6), if they did not complete RT (n = 2), and if follow-up details were missing (n = 4). Ultimately, we retrospectively reviewed medical records and tumor characteristics of 28 patients, as well as their clinical outcomes. This study was approved by the Health Institutional Review Board of Yonsei University Hospital (No. 4-2019-0608). The study was conducted in accordance with the provisions of the 1975 Declaration of Helsinki. The requirement for informed consent was waived owing to the retrospective nature of this study. All data between March 2015 and May 2019 were fully anonymized before authors accessed them.

### Treatment

All patients, except three patients who were medically ineligible due to poor performance and comorbidity, received chemotherapy using a platinum- and taxane-based regimen. Twenty-five patients began on RT administered concurrently with weekly paclitaxel (45 mg per square meter of body-surface area) via intravenous infusion over 1 hour, followed by carboplatin at an area under the plasma concentration time curve (AUC) of 2 mg/mL * minute, with a total dose of AUC * (glomerular filtration rate + 25), as an intravenous infusion over 30 minutes.

All patients underwent simulation four-dimensional CT without contrast enhancement (3-mm slice thickness) for RT planning in both initial plan and interim adaptive plan. The GTV was delineated by single radiation oncology expert with more than 30 year experience in lung cancer (C.G.L) at simulation CT with contrast enhancement, including the primary tumor and involved regional nodes (1 cm or larger in short axis, showing abnormal FDG-avidity on PET_pre_, or proven on biopsy), based on both CT and pre-RT FDG-PET/CT. The internal GTV was contoured on all-phase four-dimensional CT scans in order to reflect the effects of respiration. The clinical target volume was defined as GTV plus a 3-5-mm margin in order to include microscopic tumor extension. An additional 3-mm margin to both the internal GTV and clinical target volume was added to planning target volume (PTV1 and PTV2, respectively) based on institutional image-guidance strategies. A simultaneous integrated boost was utilized in PTV1 for 63 Gy in 30 fractions and PTV2 for 54 Gy in 30 fractions. All patients were treated with intensity-modulated RT using volumetric-modulated arc therapy (Elekta VMAT, Elekta, Stockholm, Sweden) [11]. Daily pretreatment imaging using kilovoltage cone-beam CT was performed for image-guided RT.

### ^18^F-FDG-PET/CT method

All PET_pre_ and PET_interim_ scans were performed using Discovery STE (GE Healthcare, Milwaukee, WI, USA) scanner. Every patient fasted for a minimum of 6 hours before ^18^F-FDG administration, ensuring a blood glucose level below 140 mg/dL. Patients were then injected with FDG at 5.5 MBq/kg. After allowing 45–60 minutes for tracer uptake, patients underwent PET/CT imaging along with a non-contrast low-dose CT scan for attenuation correction (30 mA, 140 kVp). Images were acquired from the base of the skull to the proximal thigh, with acquisition times of 3 minutes/bed position. The intrinsic spatial resolution of the system was approximately 5 mm (full width at half maximum) in the center of the field of view. All PET images were then reconstructed using a three-dimensional row-action maximum likelihood interactive reconstruction algorithm. All patients started RT median 16.5 days (range, 8–35 days) after the PET_pre_ scan to accurately reflect the tumor metabolism.(9) To minimize interpretation difficulty due to non-specific FDG accumulation from radiation-induced inflammation during RT, we performed a PET_interim_ scan at a median of 2 weeks (range 13–22 days) after initiation of RT [12].

### PET metrics

PET/CT images were consistently analyzed by two radiation oncology physicians (N.K. and C.G.L.) using the MIM Maestro 6.7 (MIM Software Inc., Cleveland, OH, USA). The region of interest was delineated over the primary tumor on the PET_pre_ and PET_interim_ scans using PET Edge, a semi-automatic gradient-based method validated for its superiority over manual or threshold methods [13]. This algorithm sets the contour boundary at the location where the signal gradient is highest. Then, deformable registration of delineated GTV in contrast-enhanced planning CT scans for initial and adaptive plan was performed to adjust the region of interest generated by two blinded radiation oncologists. Final region of interest for further analysis regarding PET parameter was approved by single radiation oncologist (C.G.L.). The SUV was measured in all voxels in the primary tumor region of interest. The maximum SUV (SUV_max_) was defined as the maximum decay-corrected activity concentration in the tumor/(injected dose/body weight). Since metabolic target volume or total lesion glycolysis is based on relative uncertainty compared to maximum value of SUV due to inflammation, fibrosis, or atelectasis in lung cancer, we only analyzed the SUV_max_ in the current study.

### Statistical analysis

The percentage change in each parameter between the PET_pre_ and PET_interim_ was calculated using the following equation [14]:

$$\Delta [Parameter]=\{[Parameter_{pre}-Parameter_{interim}]/Parameter_{pre}\}\times 100\%$$

Since there is limited information for universally accepted the optimal cut-off value for dynamics in PET parameters, receiver operating characteristics curve analyses regarding any failures were used to assess the cut-off threshold of SUV_max_ from PET_interim_ for identifying metabolic responders. As a reference, volumetric response was assessed based on GTV changes (ΔGTV), with a threshold of 25%, which could improve the response assessment compared to Response Evaluation Criteria in Solid Tumors [15]. Locoregional recurrence (LRR) and distant failures (DF) were defined as any first recurrence within and outside the PTV until the last follow-up, respectively. Overall survival (OS) was calculated from the day of first RT to the date of death or the last follow-up visit. Survival curves were estimated using the Kaplan-Meier method and compared using the log-rank test. Univariable analysis of LRR and DF was performed using Cox regression analysis. A multivariable analysis was not performed because no statistically significant factors were identified on univariable analysis. Sensitivity, specificity, accuracy, positive predictive value (PPV), and negative predictive value (NPV) were calculated to assess the diagnostic value of selected parameters. In addition, Delong’s test after bootstrapping 200 times was performed to compare the predictive value of selected cutoff values from parameters. The α level of 0.05 was used: a p-value <0.05 was regarded as a rejection to the null hypothesis and therefore considered statistically significant. All statistical analyses were performed using SPSS version 25.0.0 (IBM Corp., Armonk, NY) and R (version 3.6.3; R Foundation for Statistical Computing, Vienna, Austria).

## Results

### Cohort characteristics

Details of the patients’ characteristics are presented in Table 1. Males predominated (92.9%) among the entire group of 28 patients, and the median age was 73.5 years (interquartile range (IQR) 66.0–88.0). Most patients were diagnosed as having squamous cell carcinoma (64.3%), followed by adenocarcinoma (35.7%). Median primary tumor size was 4.1 cm (IQR 3.4–5.3) and more than half of the patients (82.2%) were diagnosed at stage III. The PET_interim_ was obtained approximately 11 fractions after treatment initiation, with a median dose of 23.1 Gy (IQR 23.1–24.7).

**Table 1:** Patient and treatment characteristics.

**Patient characteristics**	**N**	**%**
Age at treatment (yrs, median [IQR])	73.5	[66.0–80.0]
Sex		
Female	2	7.1
Male	26	92.9
ECOG PS		
0–1	26	92.9
2	2	7.1
Pathology		
Squamous cell carcinoma	18	64.3
Adenocarcinoma	10	35.7
Primary tumor size (cm, median [IQR])	4.1	[3.4–5.3]
≥4 cm	17	60.7
<4 cm	11	39.3
Stage		
IB—IIB	5	17.8
IIIA—IIIC	23	82.2
**Treatment characteristics**	**N**	**%**
Aim		
Definitive	28	100.0
Concurrent chemotherapy	25	89.3
Intensity-modulated radiation therapy	28	100.0
Median total dose (Gy, median [IQR])	63	[61.5–63.0]
Median fraction dose (Gy, median [IQR])	2.1	[2.1–2.2]
Fractions of RT completed before interim PET (fractions, median [range])	11	[10–14]
Dose of RT completed before interim PET (Gy, median [range])	23.1	[23.1–24.7]

*Abbreviations*: IQR, interquartile range; ECOG PS, Eastern Cooperative Oncology Group performance status; RT, radiation therapy

### Changes during RT

The median GTV_pre_ and SUV_max(pre)_ were 119.6 cc (IQR 85.7–190.6) and 15.5 (IQR 11.5–21.4), respectively. Both GTV and SUV_max_ were generally decreased on PET_interim_; median ΔGTV and ΔSUV_max_ were 23.6% (IQR 14.0–49.6%) and 32.9% (IQR 8.4–64.6%), respectively. However, four patients showed an increased GTV and another five showed increased SUV_max_. The quantitative analysis of SUV_max_ and GTV is summarized in S1 Table.

### Treatment outcomes

Median follow-up was 17.7 months (IQR 11.9–22.2). Twelve patients developed LRR, 15 patients showed DF, and 7 patients experienced both LRR and DF; 4 of them encountered with simultaneous LRR and DF as a first treatment failure. The overall 1-year LRR rate was 34.3%, while the DF rate was 36.1% for the entire cohort (S1A Fig). One-year OS and progression-free survival rates were 82.0% and 53.3%, respectively (S1B Fig).

### Prognostic factors for treatment outcomes

**Fig 1:**
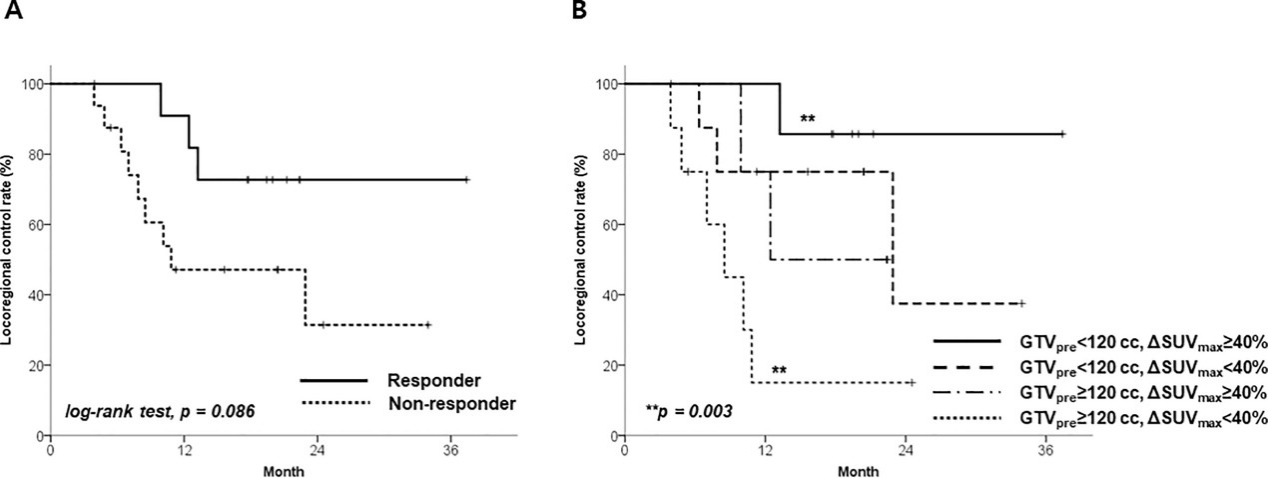
Locoregional control rate according to the SUV_max_ change: A—according to the SUV_max_ reduction rate, and B—stratified by pretreatment GTV (GTV_pre_). Responders were patients with SUV_max_ reduction rates ≥40%, whereas non-responders were those with SUV_max_ reduction rates <40%.

With an area under the receiver operating characteristics curve of 0.812 for any failures (S2 Table), a threshold of 40% was calculated as the optimal cut-off for ΔSUV_max_. With this threshold, there were 12 metabolic responders and 16 non-responders. Metabolic response based on ΔSUV_max_ of 40% demonstrated a difference in locoregional control (LRC), but this was not statistically significant for the entire cohort; 1-year LRC rate for metabolic responders (n = 12) was 90.9%, compared to 47.1% for non-responders (n = 16, Fig 1A, Table 2). However, large GTV_pre_ (≥120 cc) was identified as a poor prognostic factor for LRC on univariable analysis (HR 4.14, 95% CI 1.23–13.97; p = 0.022), whereas ΔGTV had little impact on LRC (p = 0.341). However, metabolic response showed a borderline impact on LRC, along with GTV_pre_ (Fig 1B). Metabolic responders with a small GTV_pre_ (n = 4) showed the best 1-year LRC rate, of 100%. In contrast, metabolic non-responders with a large GTV_pre_ (n = 8) showed the worst 1-year LRC rate, of 15%. There was no difference in LRC between metabolic non-responders with a small GTV_pre_ (n = 8) and metabolic responders with a large GTV_pre_ (n = 8) (1-year LRC rate 75.0% vs. 75%, p = 0.584).

**Table 2:** Predictors of locoregional recurrence identified using a Cox proportional hazards model.

		1-yr LRC	Univariable analysis		
		%	HR	95% CI	p-value
Age (yrs)	<70	57.1	ref		
	≥70	70.6	0.51	0.16–1.6	0.252
Pathology	Adenoca	57.1	ref		
	SqCCa	70.6	0.61	0.19–1.92	0.395
Size	<4 cm	77.8	ref		
	≥4 cm	58.8	1.31	0.39–4.38	0.659
T	T1-2	64.6	ref		
	T3-4	66.7	0.64	0.19–2.15	0.473
Stage	I-II	50.0	ref		
	III	68.3	0.84	0.18–3.87	0.819
Total dose	<60 Gy	0.0	ref		
	≥60 Gy	68.1	0.18	0.02–1.79	0.145
GTV_pre_	<120 cc	86.7	ref		
	≥120 cc	37.0	4.14	1.23–13.97	0.022
SUV_max(pre)_	<15	50.8	ref		
	≥15	78.6	0.97	0.9–1.05	0.447
GTV_pre_−GTV_int_	(+)	88.9	ref		
	(-)	42.9	1.39	0.22–4.28	0.093
SUVmax(pre)−SUV_max(int)_	(+)	67.1	ref		
	(-)	60.0	0.77	0.16–3.67	0.747
ΔGTV	≥ 25%	71.8	ref		
	< 25%	58.3	0.57	0.18–1.81	0.341
ΔSUV_max_	≥ 40%	90.9	ref		
	< 40%	47.1	3.02	0.8–11.32	0.101

*Abbreviations*: yr, year; LRC, locoregional control rate; HR, hazard ratio; CI, confidence interval; Adenoca, adenocarcinoma; SqCCa, squamous cell carcinoma; Gy, gray; GTV, gross tumor volume; SUV_max_, maximum standardized uptake value; X_pre_, pre-treatment value; X_int_, interim value

**Fig 2:**
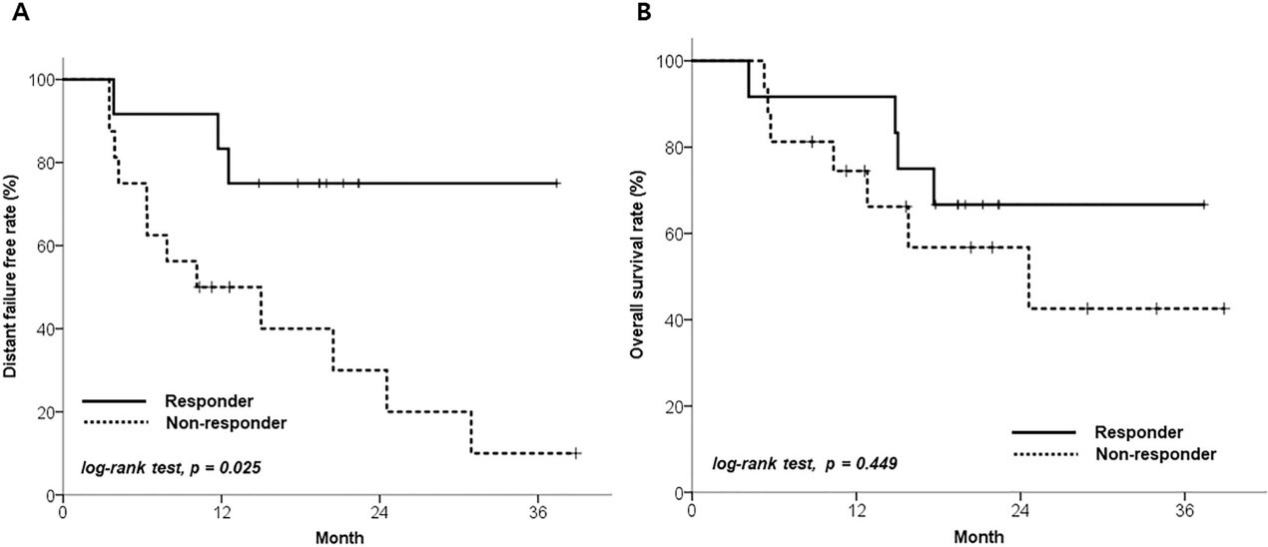
Clinical outcomes according to reduction in SUV_max_: A—Distant failure-free rate of patients, and B—overall survival rate. Responders were patients with SUV_max_ reduction rates ≥40%, whereas non-responders were those with SUV_max_ reduction rates <40%.

Patients with a favorable metabolic response showed better 1-year DF-free rate than non-responders: 83.3% for good responders and 50.0% for non-responders (p = 0.025) (Fig 2A, Table 3). However, GTV_pre_, SUV_max(pre)_, and ΔGTV were not associated with DF. Metabolic responders showed prolonged survival than non-responders, but this was not statistically significant (1-year OS rate 91.7% vs. 74.5%, p = 0.449) (Fig 2B). No statistically significant prognostic factor was found to influence OS (S3 Table).

**Table 3:** Predictors of distant failures identified using a Cox proportional hazards model.

		1-yr DFFR	Univariable analysis		
		%	HR	95% CI	p-value
Age (yrs)	<70	50.0	ref		
	≥70	72.2	0.77	0.27–2.19	0.626
Pathology	Adenoca	60.0	ref		
	SqCCa	72.2	0.87	0.30–2.50	0.800
Size	<4 cm	53.0	ref		
	≥4 cm	70.6	0.5	0.17–1.50	0.219
T	T1-2	56.3	ref		
	T3-4	75.0	0.66	0.22–1.94	0.450
Stage	I-II	40.0	ref		
	III	69.0	0.57	0.18–1.83	0.349
Total dose	<60 Gy	50.0	ref		
	≥60 Gy	64.9	0.67	0.09–5.27	0.705
GTV_pre_	<120 cc	61.9	ref		
	≥120 cc	66.7	1	1.00–1.01	0.249
SUV_max(pre)_	<15	61.5	ref		
	≥15	66.0	0.98	0.91–1.05	0.490
GTV_pre_−GTV_int_	(+)	62.2	ref		
	(-)	75.0	0.54	0.07–4.19	0.556
SUVmax(pre)−SUV_max(int)_	(+)	64.6	ref		
	(-)	60.0	1.68	0.56–5.06	0.355
ΔGTV	≥ 25%	61.5	ref		
	< 25%	65.2	0.62	0.22–1.74	0.368
ΔSUV_max_	≥ 40%	83.3	ref		
	< 40%	50.0	3.93	1.09–14.15	0.036

*Abbreviations*: yr, year; DFFR, distant failure free rate; HR, hazard ratio; CI, confidence interval; Adenoca, adenocarcinoma; SqCCa, squamous cell carcinoma; Gy, gray; GTV, gross tumor volume; SUV_max_, maximum standardized uptake value; X_pre_, pre-treatment value; X_int_, interim value

### Diagnostic tests

The diagnostic test results are presented in Table 4. Using the threshold of 40%, ΔSUV_max_ provided a sensitivity of 56.3%, specificity of 75.0%, accuracy of 64.3%, PPV of 75.0%, and NPV of 56.3% for predicting LRR. GTV_pre_ with a threshold of 120 cc was identified as a tool for predicting LRR, with a diagnostic accuracy of 71.4%. ΔSUV_max_ showed better diagnostic ability for predicting DF than GTV_pre_, with a sensitivity, specificity, and accuracy of 75.0%; PPV of 80.0%; and NPV of 69.2%. There was no statistical difference in AUC value for LRR between ΔSUV_max_ and GTV_pre_ criteria (0.656 and 0.708, p = 0.681, S2 Fig). The AUC was 0.766 and 0.603 for DF based on ΔSUV_max_ and GTV_pre_ criteria, respectively (p = 0.043, S2 Fig).

**Table 4:** Diagnostic tests for response criteria based on ΔSUV_max_ and GTV_pre_.

	Locoregional recurrence		Distant failure		Overall survival	
	Value	95% CI	Value	95% CI	Value	95% CI
**ΔSUV**_**max**_ **(40%)**						
Sensitivity	56.3	(31.9–80.6)	75.0	(53.8–96.2)	43.8	(19.4–68.1)
Specificity	75.0	(50.5–99.5)	75.0	(50.5–99.5)	66.7	(40–93.3)
False-positive rate	25.0	(5.0–49.5)	25.0	(0.5–49.5)	33.3	(6.7–60)
False-negative rate	43.8	(19.4–68.1)	25.0	(3.8–46.2)	56.3	(31.9–80.6)
Diagnostic accuracy	64.3	(46.5–82)	75.0	(59–91)	53.6	(35.1–72)
PPV	75.0	(50.5–99.5)	80.0	(59.8–100.2)	63.6	(35.2–92.1)
NPV	56.3	(31.9–80.6)	69.2	(44.1–94.3)	47.1	(23.3–70.8)
**GTV**_**pre**_ **(120cc)**						
Sensitivity	66.7	(40–93.3)	41.7	(13.8–69.6)	58.3	(30.4–86.2)
Specificity	75.0	(53.8–96.2)	37.5	(13.8–61.2)	75.0	(53.8–96.2)
False-positive rate	25.0	(3.8–46.2)	62.5	(38.8–86.2)	25.0	(3.8–46.2)
False-negative rate	33.3	(6.7–60)	58.3	(30.4–86.2)	41.7	(13.8–69.6)
Diagnostic accuracy	71.4	(54.7–88.2)	39.3	(21.2–57.4)	67.9	(50.6–85.2)
PPV	66.7	(40–93.3)	33.3	(9.5–57.2)	63.6	(35.2–92.1)
NPV	75.0	(53.8–96.2)	46.2	(19.1–73.3)	70.6	(48.9–92.3)

*Abbreviations*: CI, confidence interval; SUV_max_, maximum standardized uptake value; GTV, gross tumor volume; PPV, positive predictive value; NPV, negative predictive value.

## Discussion

In this study, we investigated the predictive value of using ^18^F-FDG-PET parameters before and during RT for predicting treatment outcomes in patients with NSCLC. Although there was a significant difference in LRC according to GTV_pre_, metabolic response showed some degree of impact based on subgroup analysis. However, changes in SUV_max_ were significantly associated with DF, and this criterion has proved its diagnostic value to predict response to RT.

Tumor burden, measured by GTV, is important in tumor control models of RT; a given dose induces a log cell kill, assuming that the larger the tumor, the more cells and, therefore, the more radiation needed for LRC [16]. Given that GTV_pre_ defined on CT was significantly associated with LRR at the RT dose (total dose of 60–63 Gy) used in the present study, it can be assumed that dose escalation is needed to achieve local control in NSCLC [17]. Secondary analysis of the RTOG 9311 study revealed that increasing GTV (>45 cm^3^) was related to poor OS and progression-free survival [18]. Several other series [19, 20] have also suggested that tumor volume is a significant prognostic factor for survival. However, a recent prospective, observational factor study of TROG 99.05 [21] found that a large primary tumor volume was not associated with poor survival, after adjusting for the effects of T and N stage. Instead, large primary tumor volume had an adverse impact on survival only within the first 18 months (comparable to the median follow-up period for the present study). In addition, changes in GTV had no impact on the treatment outcomes, and metabolic response could help stratify patients: those with a large GTV_pre_ and favorable metabolic response showed an LRC rate comparable to that of patients with a small GTV_pre_ and poor metabolic response. Several series provide evidence for a correlation between SUV and tumor cell proliferation [22]. An early reduction in FDG uptake during treatment can predict tumor response. In addition, SUV_max_ represents the enhanced tapping of ^18^F-FDG into the tumor cells, due to biological mechanisms, tumor aggressiveness, and hypoxia [23].

Owing to the heterogeneity of patient populations with NSCLC at an advanced stage, there is no concrete evidence regarding the prognostic value of PET_pre_. A recent meta-analysis of 13 studies with 1474 patients demonstrated that high SUV_max(pre)_ in the primary tumor was associated with reduced survival [24]. Another meta-analysis of 36 studies on 5807 patients with surgically treated NSCLC also identified SUV_max(pre)_ as a prognostic factor for disease-free survival, with an HR of 1.52 (95% CI 1.16–2.00). However, the retrospective study by Hoang et al. [25] with a homogeneous population did not find a correlation between metabolic parameters on PET_pre_ and survival, which is consistent with the findings of the present study.

Discriminating non-responders from responders can help physicians to avoid unnecessary toxicity in patients expected to have a poor prognosis, by early interruption of ineffective therapy. Because changes in FDG uptake were associated with tumor shrinkage, PET_interim_ can also help physicians decide when to modify the RT plan, with PTV modification or dose escalation. Several series with various sample sizes (10–77 patients) have shown the prognostic value of PET_interim_ in patients with NSCLC treated with RT [26, 27] and in those with other solid tumors [28, 29]. And secondary analysis of ESPATUE study revealed that remaining SUVmax in the primary tumor after induction chemotherapy was associated with survival and freedom from extracranial progression in consistent to the current study [30]. Furthermore, a recent meta-analysis of 21 studies on 627 patients reported PET_interim_ as a promising tool for the early judgment of treatment [12]. However, because most of these studies were retrospective and examined multiple outcomes, concerns around the statistics include the fact that there were multiple comparisons and selective reporting of endpoints. More importantly, definite criteria or standard parameters have not yet been determined, and prognostic metrics range from SUV_max_ [27] and ΔSUV_max_ [31] to total lesion glycolysis [32] and metabolic tumor volume [14]. In our series, ΔSUV_max_ was associated with DF and LRR, suggesting that this parameter helps to stratify patients. Metabolic response based on ΔSUV_max_ was not significantly associated with LRC on univariable analysis, possibly due to the lack of statistical power.

However, SUV as a semiquantitative index has limitations owing to poor reproducibility [24], making it difficult to adopt a threshold among different centers. In place of the SUV value itself, we calculated a cut-off value for ΔSUV_max_ (a 40% reduction), which was predictive of both LRR and DF. Criteria for the relative change in SUV_max_ can be a tool for predicting early treatment response in the same institution, which, in turn, can minimize the issue of variability and enhance the prognostic value of this metabolic parameter.

Early response appears to be an indicator of tumor biology and a predictor of the likelihood of treatment failure. Thus, the assessment of early response makes it easier to identify poor responders who are eligible for the intensification or modification of treatment, instead of continuation of the initial treatment (the so-called ^18^F-FDG-PET/CT-guided treatment algorithm). A recent phase II trial proved that adaptive RT with escalated doses accompanied by PET_interim_ is feasible and results in favorable LRC [33]. A further ongoing clinical trial (RTOG 1106) is examining adaptive RT with dose escalation for FDG-avid tumors on PET_interim_. Another promising area of research that needs further prospective trials is the early switching of systemic chemotherapy in patients with a small decrease in SUV_max_. Recently, there are several on-going trials in other solid tumors investigating the role of immune checkpoint blockade stratified by PET parameters (NCT 03829007, NCT 03853187, NCT 02760225).

Our study had several limitations. First, as a retrospective analysis, the results should be interpreted with caution. Although we have analyzed an optimal cut-off value for SUV_max_, we used a median value of GTV and 25% criteria for ΔGTV as previously reported to minimize statistical overfitting. Optimal threshold could be derived from further investigation with large number of patients and it should be externally validated. Second, there are inherent biases since this study was carried out in a single institution. However, our analysis was strengthened using consistent modern ^18^F-FDG-PET/CT, imaging analyses, chemotherapy regimen, and RT techniques. Other limiting factors include possible inflammatory changes caused by irradiation, which may mimic changes in tumor glucose metabolism associated with treatment. We evaluated the PET_interim_ at 2 weeks after initiation of RT to minimize the overlapping of inflammation and residual tumor [12]. In addition, there is a possibility of overestimation of changes in SUV, because of the partial-volume effect; tumor reduction may underestimate the FDG uptake. Lastly, lack of a univocal parameter remains a challenge in dealing with the metabolic parameters as a universal prognostic or predictive factor. Although FDG uptake is generally used as a parameter to reflect the proportion of viable tumor cells, new tracers are now available for specifically detecting apoptosis and proliferation to provide a highly accurate prediction of treatment response.

## Conclusions

We could cautiously assume that response criteria based on changes in SUV_max_ during RT could be useful for identifying responders to current treatment among patients with NSCLC. The optimal management of poor responders identified on PET_interim_ remains to be determined. Furthermore, a prospective study to confirm the efficacy of ^18^F-FDG-PET/CT-guided algorithms in patients with NSCLC is warranted.

## Supporting information

S1 Fig Clinical outcomes of the entire cohort. A—locoregional recurrence (LRR) and distant failure (LR) rate, and B—overall survival (OS) and progression-free survival (PFS) rate.
(DOCX)

S2 Fig Receiver operating characteristic curve according to ΔSUV_max_ and GTV_pre_ crietria for A- locoregional recurrence, and B–distant failure. (DOCX)

S1 Table Quantitative parameters on the pretreatment and interim PET scan. (DOCX)

S2 Table Diagnostic tests for response criteria of ΔSUV_max_ 40% in treatment failure. (DOCX)

S3 Table Predictors of overall survival identified using a Cox proportional hazards model. (DOCX)

S1 Data (XLSX)
