# Supplementary material for: Predictive value of interim 18F-FDG-PET in patients with non-small cell lung cancer treated with definitive radiation therapy
Source: PLoS One. 2020 Jul 20;15(7):e0236350. doi: 10.1371/journal.pone.0236350 (PMC7371172; doi:10.1371/journal.pone.0236350)

S1 Fig. Clinical outcomes of the entire cohort: A – locoregional recurrence (LRR) and distant failure (LR) rate, and B – overall survival (OS) and progression-free survival (PFS) rate.


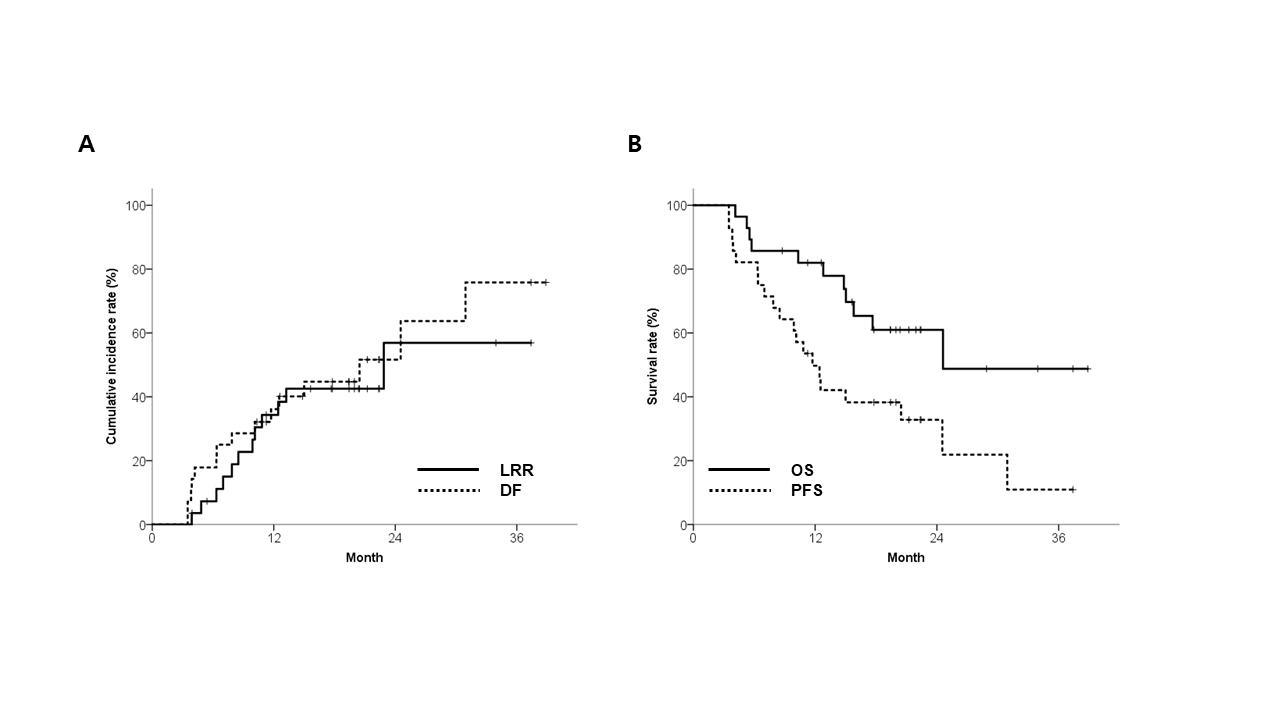

Supplement: S1 Fig — A—locoregional recurrence (LRR) and distant failure (LR) rate, and B—overall survival (OS) and progression-free survival (PFS) rate. (DOCX) [file pone.0236350.s001.docx]
