# Supplementary material for: Predictive value of interim 18F-FDG-PET in patients with non-small cell lung cancer treated with definitive radiation therapy
Source: PLoS One. 2020 Jul 20;15(7):e0236350. doi: 10.1371/journal.pone.0236350 (PMC7371172; doi:10.1371/journal.pone.0236350)

S2 Fig. Receiver operating characteristic curve according to ΔSUV_max_ and GTV_pre_ crietria for A- locoregional recurrence, and B – distant failure.


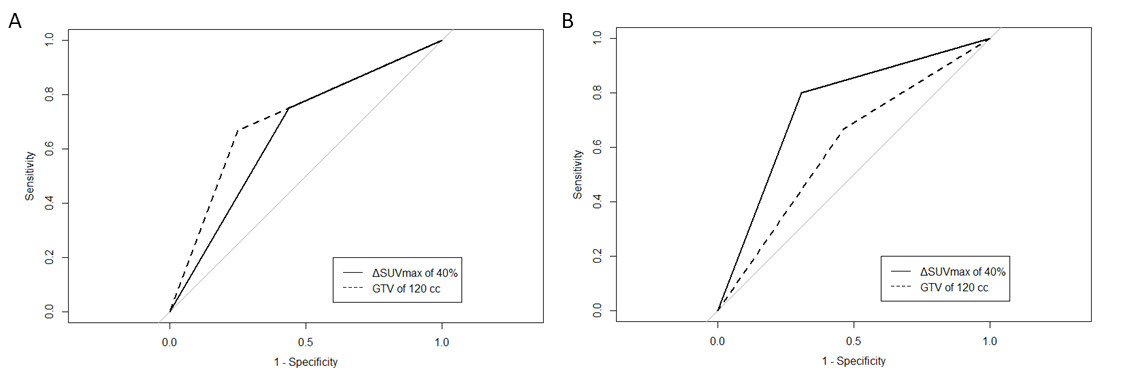

Supplement: S2 Fig — (DOCX) [file pone.0236350.s002.docx]
