# Supplementary material for: Predictive value of interim 18F-FDG-PET in patients with non-small cell lung cancer treated with definitive radiation therapy
Source: PLoS One. 2020 Jul 20;15(7):e0236350. doi: 10.1371/journal.pone.0236350 (PMC7371172; doi:10.1371/journal.pone.0236350)
Supplement: S1 Table — (DOCX) [file pone.0236350.s003.docx]

**Supplementary Table 1. Quantitative parameters on the pretreatment and interim PET scan**

|  | **Pretreatment PET** | |  | **Interim PET** | |  | **% of decrease** | |
| --- | --- | --- | --- | --- | --- | --- | --- | --- |
|  | **Median** | **IQR** |  | **Median** | **IQR** |  | **Median** | **IQR** |
| GTV (cc) | 116.6 | [85.7;190.6] |  | 78.1 | [52.2;111.4] |  | 23.6 | [14.0;49.6] |
| SUV_max_ | 15.5 | [11.5;21.4] |  | 8.5 | [6.1;11.0] |  | 32.9 | [8.4;64.6] |

*Abbreviations:* IQR, interquartile range; GTV, gross tumor volume; SUV_max_, maximum standardized uptake value
