# Supplementary material for: Predictive value of interim 18F-FDG-PET in patients with non-small cell lung cancer treated with definitive radiation therapy
Source: PLoS One. 2020 Jul 20;15(7):e0236350. doi: 10.1371/journal.pone.0236350 (PMC7371172; doi:10.1371/journal.pone.0236350)
Supplement: S2 Table — (DOCX) [file pone.0236350.s004.docx]

**Supplementary Table 2. Diagnostic tests for response criteria of** **ΔSUV_max_ 40%**

|  | Any failures | |
| --- | --- | --- |
|  | Value | 95% CI |
| **ΔSUV_max_ (40%)** |  |  |
| Sensitivity | 75 | (50.9-86.8) |
| Specificity | 87.5 | (47.3-99.7) |
| False-positive rate | 12.5 | (3.2-52.7) |
| False-negative rate | 25 | (8.7-49.1) |
| Diagnostic accuracy | 78.6 | (59.0-91.7) |
| PPV | 93.8 | (69.8-99.8) |
| NPV | 58.3 | (27.7-84.8) |

*Abbreviations:* CI, confidence interval; SUV_max_, maximum standardized uptake value; PPV, positive predictive value; NPV, negative predictive value.
