# Supplementary material for: Predictive value of interim 18F-FDG-PET in patients with non-small cell lung cancer treated with definitive radiation therapy
Source: PLoS One. 2020 Jul 20;15(7):e0236350. doi: 10.1371/journal.pone.0236350 (PMC7371172; doi:10.1371/journal.pone.0236350)
Supplement: S3 Table — (DOCX) [file pone.0236350.s005.docx]

**Supplementary Table 3. Predictors of overall survival identified using a Cox proportional hazards model**

|  |  | **1-yr OS** | **Univariable analysis** | | | | |
| --- | --- | --- | --- | --- | --- | --- | --- |
|  |  | **%** | **HR** | **95% CI** | | | **p-value** |
| Age (yrs) | <70 | 70.0 | ref |  |  |  |  |
|  | ≥70 | 88.5 | 0.73 | 0.22 | - | 2.43 | 0.608 |
| Pathology | Adenoca | 70.0 | ref |  |  |  |  |
|  | SqCCa | 88.5 | 0.57 | 0.17 | - | 1.89 | 0.362 |
| Size | <4 cm | 72.7 | ref |  |  |  |  |
|  | ≥4 cm | 88.2 | 0.47 | 0.14 | - | 1.56 | 0.219 |
| T | T1-2 | 81.3 | ref |  |  |  |  |
|  | T3-4 | 83.3 | 0.72 | 0.21 | - | 2.5 | 0.605 |
| Stage | I-II | 80.0 | ref |  |  |  |  |
|  | III | 82.6 | 1.99 | 0.25 | - | 15.57 | 0.513 |
| Total dose | <60 Gy | 50.0 | ref |  |  |  |  |
|  | ≥60 Gy | 84.4 | 0.3 | 0.03 | - | 2.67 | 0.277 |
| GTV_pre_ | <120 cc | 93.8 | ref |  |  |  |  |
|  | ≥120 cc | 66.7 | 1.01 | 1.00 | - | 1.01 | 0.007 |
| SUV_max(pre)_ | <15 | 69.2 | ref |  |  |  |  |
|  | ≥15 | 83.3 | 0.98 | 0.91 | - | 1.06 | 0.685 |
| GTV_pre_ – GTV_int_ | (+) | 83.1 | ref |  |  |  |  |
|  | (-) | 75.0 | 0.74 | 0.09 | - | 5.83 | 0.772 |
| SUV_max(pre)_ – SUV_max(int)_ | (+) | 78.3 | ref |  |  |  |  |
|  | (-) | 100.0 | 0.36 | 0.05 | - | 2.86 | 0.335 |
| ΔGTV | ≥ 25% | 84.6 | ref |  |  |  |  |
|  | < 25% | 79.4 | 0.99 | 0.3 | - | 3.25 | 0.987 |
| ΔSUV_max_ | ≥ 40% | 91.7 | ref |  |  |  |  |
|  | < 40% | 74.5 | 1.62 | 0.46 | - | 5.67 | 0.453 |

*Abbreviations:* yr, year; OS, overall survival; HR, hazard ratio; CI, confidence interval; Adenoca, adenocarcinoma; SqCCa, squamous cell carcinoma; Gy, gray; GTV, gross tumor volume; SUV_max_, maximum standardized uptake value; X_pre,_ pre-treatment value; X_int_, interim value
